# Do College Freshmen Who Engage More in Online Social Comparison Tend to Be More Confused About Themselves? The Roles of Rumination and Self-Compassion

**DOI:** 10.3390/bs15070849

**Published:** 2025-06-24

**Authors:** Sujie Kang, Qian Gu, Wen Qin, Sanming Liu, Yukang Xue, Qishan Zheng, Chuanhua Gu, Yuqi Cao

**Affiliations:** 1 Key Laboratory of Adolescent Cyberpsychology and Behavior (CCNU), Ministry of Education, Wuhan 430079, China; ksj@mail.hzau.edu.cn (S.K.); qwen@mails.ccnu.edu.cn (W.Q.); zhengqishan23@163.com (Q.Z.); 2 Key Laboratory of Human Development and Mental Health of Hubei Province, School of Psychology, Central China Normal University, Wuhan 430079, China; 3 Mental Health Education Center, Huazhong Agricultural University, Wuhan 430070, China; lsm@mail.hzau.edu.cn; 4 School of Educational Science, Liaocheng University, Liaocheng 252059, China; guqian2023@163.com; 5 Department of Educational and Counseling Psychology, University at Albany, SUNY 1400 Washington Avenue, Albany, NY 12222, USA; yxue2@albany.edu; 6 School of Humanities, Political Science and Law, Henan University of Engineering‌, Zhengzhou 451191, China

**Keywords:** online social comparisons, self-concept clarity, rumination, self-compassion, college freshmen

## Abstract

Online social comparisons play a vital role in adolescents and young adults’ self-development. This study extended research on the influence of online social comparisons on self-concept clarity among college freshmen. This study investigated the mediating role of rumination in the relationship between online social comparisons and self-concept clarity among college freshmen and further examined the moderating effect of self-compassion on this mediational pathway. A sample of 975 Chinese university freshmen were recruited to complete the Online Social Comparisons Scale, Self-Concept Clarity Scale, Rumination Scale and Self-Compassion Scale. The results indicated that among college freshmen, online social comparisons can negatively impact self-concept clarity both directly and indirectly through rumination. Self-compassion moderated the effect of rumination on self-concept clarity. Compared to college freshmen with high self-compassion, those with low self-compassion showed a steeper decline in self-concept clarity as rumination increased. This study not only uncovers the psychological mechanisms through which online social comparison damages self-concept clarity but also provides empirical support for universities to develop targeted psychological health intervention programs based on self-compassion.

## 1. Introduction

Self-concept clarity (SCC) refers to the extent to which individuals’ beliefs about themselves are clearly defined, internally consistent, and stable over time (Campbell et al., 1996). It is considered a structural quality of the self and is closely related to identity development and psychological functioning. People with high self-concept clarity possess a coherent and stable sense of self, which contributes to emotional regulation and resilience (Bigler et al., 2001; Campbell, 1990). Conversely, individuals with low self-concept clarity often experience confusion and psychological distress due to fragmented self-views (Van Dijk et al., 2014). Self-concept clarity has been shown to correlate positively with subjective well-being (Xiang et al., 2023), grit (S. Chen et al., 2024), and meaning in life (S. Chen et al., 2024), and negatively with anxiety and depression (Bigler et al., 2001; Van Dijk et al., 2014).

### 1.1. Online Social Comparisons and Self-Concept Clarity

Social comparison is a crucial factor in the formation and development of an individual’s self-concept (Festinger, 1954). According to Festinger’s (1954) classic social comparison theory, humans possess an inherent drive to evaluate their own viewpoints and abilities in order to achieve accurate self-awareness. In the absence of objective, non-social benchmarks, individuals reduce uncertainty by comparing themselves to others. Festinger further proposed that to obtain the most accurate self-evaluation, people tend to compare themselves with others who are similar to them in relevant attributes. Through such comparisons with stable reference groups, individuals gradually form and confirm beliefs about “who I am,” which constitute the foundation of their self-concept. As initially conceptualized by Festinger (1954), the theory distinguishes between two primary directions of comparison. Upward social comparison occurs when individuals compare themselves to others who are perceived as superior or better off, which can elicit negative feelings like envy but may also serve as inspiration. Conversely, downward social comparison involves comparing oneself to those who are perceived as less fortunate, a strategy that is often used for self-enhancement to boost self-esteem and positive affect (Wills, 1981).

With the widespread use of online social networks, social comparison theory has evolved in response to new contexts, particularly with the rise in online social interactions. Modern research has expanded the definition of social comparison to include not only comparisons in physical environments but also comparisons occurring through online platforms such as social media (Vogel et al., 2014). In this context, online social comparisons refer to the process of individuals comparing themselves with others through digital media, often involving curated, idealized portrayals of others’ lives. Compared to offline comparisons, online social comparisons are characterized by immediacy and diversity. The basis of such comparisons often consists of others’ carefully curated and idealized “highlight moments,” which are saturated with a positivity bias (Vogel et al., 2014; Verduyn et al., 2020). This continuous exposure to the success, appearance, and happy experiences of others makes users highly susceptible to engaging in substantial upward social comparison. A series of meta-analyses have confirmed that online social comparisons are core processes that undermine users’ well-being and self-evaluation, with significant negative impacts on mental health (such as depression and anxiety) and self-perception (such as the state of an individual’s self-esteem and body image) (Marciano et al., 2024; McComb et al., 2023; Yang et al., 2018).

Therefore, we hypothesize that online social comparisons may undermine the consistency, clarity, and stability of the self-concept. Specifically, online social comparisons tend to focus on dimensions such as physical attractiveness (e.g., body shape, facial appearance) and lifestyle and success (e.g., academic achievements, career development, travel experiences, and social life) (Fardouly & Vartanian, 2016; Vogel et al., 2014). When individuals are exposed to continuously emerging and divergent standards of idealized appearances and successful lifestyles online, the reference points that they use for self-evaluation become confusing and increasingly unattainable. As a result, individuals may struggle to form a coherent and unified standard for the self, leading to internal conflicts in beliefs (e.g., “I study hard, but is my life meaningful compared to those who travel the world?”), which in turn diminishes the clarity and internal consistency of the self-concept.

Furthermore, online social comparisons can lead to instability in self-evaluation, where individuals’ self-perceptions may fluctuate dramatically in response to a single photograph or status update. Existing studies provide preliminary support for this reasoning. For example, Orben et al. (2024) conducted a systematic review of research on social media use and adolescent mental health, concluding that online social comparisons negatively affect the development of self-concept in adolescents. In addition, empirical evidence from Niu et al. (2024) demonstrated that online upward social comparison impairs self-concept clarity among Chinese young adults.

This issue may be particularly salient for first-year university students, who are typically in the critical developmental stage of emerging adulthood (Arnett, 2000). During this period, identity exploration is at its peak, and the self-concept is inherently more malleable and susceptible to external feedback. Therefore, we hypothesize that online social comparisons negatively predict self-concept clarity among first-year university students.

### 1.2. The Mediating Role of Rumination

From the perspective of social cognitive theory, research on social comparison highlights that understanding how people think during the comparison process—such as when they engage in rumination—is key to understanding how these comparisons affect their self-concept (Wood, 1996). Rumination, a form of self-focused attention or introspection, is closely associated with psychological threat, uncertainty, and perceived inadequacy. It often arises after negative events and is characterized by persistent, repetitive thinking about one’s distress and its possible causes and consequences (Nolen-Hoeksema et al., 2008; Robinson & Alloy, 2003; Trapnell & Campbell, 1999). A substantial body of research has delineated rumination as a maladaptive cognitive style that is both passive and repetitive, involving abstract, evaluative processing of negative emotional content (Watkins, 2008). Notably, rumination can be differentiated into two subtypes: brooding—a moody pondering that is considered particularly detrimental—and reflective pondering, which involves problem-focused self-analysis but may still prolong a negative mood (Treynor et al., 2003). Over time, the tendency to ruminate becomes a stable cognitive habit that sensitizes individuals to future stressors and amplifies negative affect (Nolen-Hoeksema et al., 2008).

In the context of social media, rumination has emerged as a key mechanism through which social comparison exerts its psychological impact. Most prior research on social networking sites (SNSs) has found that online social comparison—especially upward comparison—poses a threat to users’ self-esteem (Brandenberg et al., 2018; Stapleton et al., 2017; Ouwerkerk & Johnson, 2016). According to the Self-Discrepancy Theory (Higgins, 1987), when there is a significant gap between an individual’s “actual self” and their “ideal self” or “ought self,” their self-esteem is adversely affected. On social media, users are constantly exposed to carefully curated and beautified “highlight moments” of others, and these idealized images are inadvertently internalized as benchmarks for self-evaluation. This perceived discrepancy amplifies the gap between the actual self and the ideal self, triggering negative emotions such as inadequacy, jealousy, and disappointment. These emotions directly impact an individual’s self-esteem (Ouwerkerk & Johnson, 2016). Based on the stress reactivity and stress rumination model (Nolen-Hoeksema et al., 2008), when individuals perceive a threat to their self-esteem, they are more prone to engage in ruminative thinking. Empirical studies have supported this model. For instance, Feinstein et al. (2013) found that Facebook-based social comparison predicted increased rumination. Therefore, we hypothesize that online social comparisons may increase individuals’ rumination.

Furthermore, rumination may undermine self-concept clarity through several mechanisms. First, rumination can amplify and solidify negative self-perceptions (Nolen-Hoeksema et al., 1994). When individuals experience feelings of inadequacy due to online social comparisons, rumination leads them to repeatedly dwell on perceived “failures” or “deficiencies.” This persistent negative self-focus reinforces a one-sided, flawed self-image and makes it difficult for individuals to recognize and believe in their positive attributes, thereby directly impairing the clarity of their self-concept.

Second, the development of a clear self-concept requires individuals to actively process life experiences and integrate them into a coherent self-narrative. However, rumination is known to impair psychological functioning by fostering a cycle of negative thinking, low motivation, and ineffective coping. This cognitive impasse may hinder individuals from resolving internal conflicts (e.g., between the “ideal self” and the “actual self”) or from deriving a stable and coherent sense of self from their experiences. As a result, rumination weakens the internal consistency and stability of the self-concept (Aldao et al., 2010; Lyubomirsky & Tkach, 2003). This dysfunctional pattern is particularly damaging during adolescence and emerging adulthood, where it has been identified as a risk factor for disrupted self-development. Studies have shown that rumination is negatively correlated with self-concept clarity, self-acceptance, and identity integration (Diehl & Hay, 2011; Harrington & Loffredo, 2010; Şimşek, 2013; Willis & Burnett, 2016). Therefore, this study hypothesizes that rumination mediates the relationship between online social comparisons and self-concept clarity.

### 1.3. The Moderating Role of Self-Compassion

The majority of existing research has reported negative associations between online social comparisons and self-esteem or subjective well-being (Yang et al., 2018; Lim & Yang, 2015; Vogel et al., 2014; Weinstein, 2017).

However, some studies have also identified positive effects, such as the elicitation of benign envy and improvements in social self-evaluation (Meier et al., 2020; Meier & Schäfer, 2018; Ding, 2017). It can be hypothesized that there may be an additional variable moderating the complex relationship between online social comparisons and psychological outcomes. The current literature on the psychological implications of online social comparisons highlights the importance of individual differences, including, but not limited to, demographic factors such as gender and age, as well as psychological traits like personality—for example, for respondents with high Goal-Drive Persistence, online social comparisons have a positive association with eudaimonic well-being (Nesi & Prinstein, 2015; Kim et al., 2018; Gerson et al., 2016). Given the critical role of individual differences in shaping the relationship between internet use and psychological outcomes, there is an urgent need for further research on how individual variables influence the impact of online social comparisons on self-concept clarity.

Self-compassion refers to the ability to bring a sense of loving kindness, connection, and care into one’s experience of suffering (Neff, 2003, 2023). It encompasses three main components: self-kindness versus self-judgment, common humanity versus isolation, and mindfulness versus over-identification (Neff, 2003). Individuals with high self-compassion treat themselves with warmth and understanding when confronted with failure or inadequacy, acknowledge that such experiences are part of being human, and maintain a balanced awareness of their emotions without over-reacting.

Compared to self-esteem, self-compassion is considered a more stable and unconditional source of self-worth, as it does not depend on social comparison, performance, or validation (Neff & Vonk, 2009). Instead, it serves as a protective psychological resource that buffers against the negative effects of failure, rejection, and self-criticism. Meta-analytic evidence shows that self-compassion is strongly associated with indicators of psychological well-being, including greater life satisfaction, emotional resilience, and lower levels of anxiety and depression (MacBeth & Gumley, 2012).

Importantly, emerging research suggests that self-compassion plays a crucial role in shaping self-concept clarity, which refers to the extent to which one’s self-beliefs are clearly and confidently defined, internally consistent, and stable over time (Campbell et al., 1996).

Chew and Ang (2023) found that self-compassion can help individuals respond more kindly to stressful situations, thereby improving functioning and promoting life satisfaction. Miyagawa (2024) further demonstrated that enhancing self-compassion in response to negative events can help individuals maintain self-concept clarity and become more open to self-change. From a cognitive–affective regulation perspective, self-compassion is regarded as an adaptive emotion regulation strategy (Allen & Leary, 2010). It helps individuals reinterpret negative experiences in a constructive light, thereby reducing the cognitive burden of rumination and the emotional volatility that often undermines a stable self-view. Leary et al. (2007) showed that individuals with a high level of self-compassion responded to distressing events (e.g., failure or embarrassment) with less emotional reactivity and more balanced self-reflection.

Thus, it is expected that self-compassion may buffer the detrimental effects of rumination that are caused by online social comparisons. When individuals with high self-compassion engage in social comparison, they may still experience some degree of self-evaluation, but they are less likely to engage in harsh self-criticism or identity confusion. Instead, their compassionate stance allows them to acknowledge shortcomings without over-identifying with them, facilitating self-acceptance and integration—core components of self-concept clarity.

Therefore, this study hypothesizes that self-compassion moderates the relationship between rumination and self-concept clarity, potentially attenuating the negative cognitive effects of social comparison and promoting a more integrated and coherent self-view.

### 1.4. The Current Study

Existing research on online social comparison have primarily focused on its role in the relationship between social media use and individuals’ self-perception and well-being (Liu et al., 2017; Vogel et al., 2015; Wang et al., 2017; Yang et al., 2018; Yoon et al., 2019). However, how online social comparison specifically affects the structural dimensions of the self-concept—particularly self-concept clarity—has not yet been sufficiently explained. Investigating this underlying mechanism is essential, as self-concept clarity plays a critical role in individuals’ psychological well-being, identity development, and ability to cope with life’s challenges. Clarifying the internal mechanism through which online social comparisons influence self-concept clarity would significantly enhance our understanding of how individuals develop self-knowledge in digital social environments.

Moreover, college freshmen often experience self-uncertainty during their transition into new roles and unfamiliar environments that are filled with unknowns. In such a transitional phase, these students are more prone to engaging in self-evaluation through online social comparisons (Lockwood et al., 2012). Therefore, it is meaningful to examine whether and how online social comparisons affect the self-concept clarity of college freshmen, with the aim of informing the development of effective intervention strategies.

**Figure 1:**
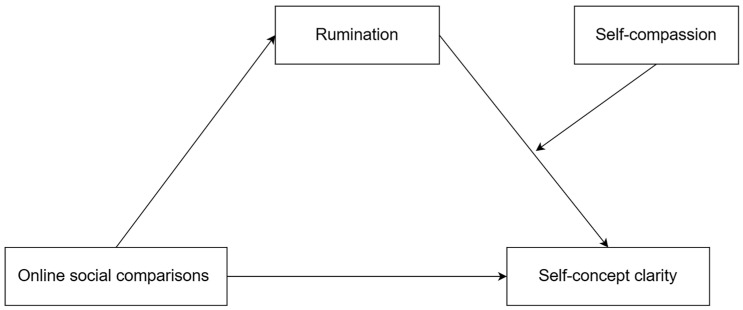
Theoretical model of the current research.

In light of this, the present study proposes a moderated mediation model to examine the mediating role of rumination in the relationship between online social comparison and self-concept clarity among college freshmen, as well as the moderating role of self-compassion in this process (see Figure 1). Based on the aforementioned literature review, the following hypotheses are proposed:

**Hypothesis** **1.** *Online social comparisons of college freshmen negatively predict self-concept clarity.*

**Hypothesis** **2.** *Rumination mediates the association between online social comparisons and self-concept clarity.*

**Hypothesis** **3.** *The mediating effect of rumination between online social comparisons and self-concept clarity (the second half path) is moderated by self-compassion.*

## 2. Materials and Methods

### 2.1. Participants and Procedure

Participants were recruited using random cluster sampling from three colleges in Hubei Province, China. In 2023, online questionnaires were distributed to these first-year students via the Questionnaire Star platform, with participation being based on voluntary responses. A total of 1095 freshman students participated in this study, and 120 students were excluded due to their failure to answer the attention check items and/or because their answering time was less than 180 s. This left a final sample of 975 participants (43.18 percent males) with an average age of 18.15 years old (*SD* = 0.635 years old, ranging from 16 to 22 years old). In addition, a power analysis using the R 4.5.0 package WebPower (Zhang & Yuan, 2018) was performed to estimate the required sample size. To provide adequate power (95%), a sample of 146 examinees was required to detect a medium effect size of *f*^2^ = 0.15.

All procedures performed in this study, which involved human participants, were in accordance with the ethical standards of the institutional and/or national research committee and the 1964 Helsinki Declaration and its later amendments or comparable ethical standards. This study was approved by the Ethics Review Board of the School of Psychology at Central China Normal University (Protocol Number: CCNU-IRB-202309032b). With standardized instructions, all participants were allotted 10–15 min during class to complete the questionnaires.

### 2.2. Measures

#### 2.2.1. Online Social Comparisons

The measurement of online social comparison was based on the Iowa–Netherlands Comparison Orientation Measure (Gibbons & Buunk, 1999), adapted and localized by Bai et al. (2013). This scale restricts the environment of comparison to social media contexts such as QQ Space or WeChat. It includes two subscales, online upward social comparison and online downward social comparison, each consisting of 6 items. Participants rated all the items on a five-point scale (1 = strongly disagree, 5 = strongly agree). In the present study, Cronbach’s *α* was 0.903 for the total scale. The Cronbach’s α values for the subscales of online upward social comparison and online downward social comparison were 0.916 and 0.911, respectively.

#### 2.2.2. Self-Concept Clarity

Self-concept clarity was assessed using the Chinese version of the Self-Concept Clarity Scale (Campbell et al., 1996), adapted and translated by Liu et al. (2017). The scale consists of 12 items rated on a five-point Likert scale (1 = strongly disagree, 5 = strongly agree). A sample item is as follows: “Generally speaking, I have a clear understanding of what kind of person I am.” In the present study, the scale demonstrated good internal consistency, with a Cronbach’s α of 0.853.

#### 2.2.3. Rumination

For rumination, we selected the Chinese version of the Nolen-Hoeksema Rumination Scale (Nolen-Hoeksema & Morrow, 1991), translated by Han and Yang (2009). The questionnaire comprises 22 items that assess three dimensions of rumination: depressive symptom rumination, brooding, and reflective pondering. Symptom rumination specifically denotes the individual’s introspective focus on depressive symptoms. Therefore, two dimensions of brooding and reflective pondering were selected in this study, with a total of 10 items. A four-point scale (1 = strongly disagree, 4 = strongly agree) was adopted. In the present study, the Cronbach’s *α* values for the subscales of brooding and reflective pondering were 0.745 and 0.842.

#### 2.2.4. Self-Compassion

For self-compassion, we used the Self-Compassion Scale (Neff, 2003), adapted and localized by J. Chen et al. (2011). This scale comprises 26 items and is combined with six subscales, with responses ranging from 1 (almost never) to 5 (almost always). Sample items include “When I’m down and out, I remind myself that there are lots of other people in the world feeling like I am”. In the present study, Cronbach’s *α* was 0.895.

### 2.3. Statistical Analysis

Data were prepared and analyzed using IBM SPSS 27.0. The moderated mediation model was tested by multiple regression using the SPSS Macro PROCESS 4.0 (model 4; model 14) with 5000 bootstrap resamples (Hayes, 2013). All the analyses were performed after controlling for covariates (gender, age). This survey was an online survey with no missing values. And the distribution of the main variables was generally consistent with a normal distribution.

## 3. Results

### 3.1. Preliminary Analyses

Harman’s single-factor test was conducted in this study to measure common method bias (Podsakoff et al., 2003). The results revealed that there were ten factors with eigenvalues greater than 1. The first factor accounted for only 22.48% of the variance, below the critical threshold of 40%. Therefore, it can be concluded that there is no significant issue of common method bias in this study.

Bivariate associations between variables are reported in Table 1. The correlational relationships among variables support further analyses.

### 3.2. Testing for Moderated Mediation Effects

According to the recommendations of Wen and Ye (2014), the research variables were standardized. With gender and age being controlled, self-concept clarity was set as the dependent variable, online social comparisons as the independent variable, and rumination as the mediator. Model 4 was employed to test the mediation effect. The results demonstrated that online social comparison significantly predicted self-concept clarity (*β* = −0.30, *p* < 0.001). After introducing rumination as a mediator, this predictive effect remained significant (*β* = −0.17, *p* < 0.001). Furthermore, online social comparison positively predicted rumination (*β* = 0.31, *p* < 0.001), while rumination negatively predicted self-concept clarity (*β* = −0.44, *p* < 0.001). The mediation effect was estimated at −0.13, with a 95% BootCI of [−0.17, −0.10] that excluded 0. The indirect effect accounted for 43.33% of the total effect, indicating that rumination partially mediated the relationship between online social comparisons and self-concept clarity.

Subsequently, a moderated mediation model was constructed with self-compassion as the moderator. Based on the hypothesized model, Model 14 was selected for analysis. The regression results showed that rumination significantly negatively predicted self-concept clarity, and the interaction term between rumination and self-compassion significantly predicted self-concept clarity (*β* = 0.05, *t* = 2.40, *p* < 0.05; see Table 2). This suggests that the latter path of rumination’s mediating effect between online social comparisons and self-concept clarity was moderated by self-compassion. To further examine this moderation, a simple effect analysis was conducted by categorizing self-compassion into high and low groups (±1 SD from the mean). The results revealed that compared to college freshmen with high self-compassion (*Slope* = −0.30, *t* = −9.28, *p* < 0.001), those with low self-compassion showed a steeper decline in self-concept clarity as rumination increased (*Slope* = −0.40, *t* = −10.86, *p* < 0.001). The simple slope analysis figure can be found in Supplementary Materials Figure S1.

Two separate moderated mediation models were also created to examine the impacts of online upward social comparison and online downward social comparison on self-concept clarity, respectively. Firstly, the impact of online upward social comparison on self-concept clarity was examined. This study set self-concept clarity as the dependent variable, online upward social comparison as the independent variable, and rumination as the mediator, using Model 4 to test the mediating effect. The results showed that online upward social comparison significantly predicted self-concept clarity (*β* = −0.26, *p* < 0.001). After introducing rumination as a mediator, this predictive effect remained significant (*β* = −0.10, *p* < 0.01). Additionally, online upward social comparison positively predicted rumination (*β* = 0.36, *p* < 0.001), and rumination negatively predicted self-concept clarity (*β* = −0.45, *p* < 0.001). The mediation analysis revealed a mediation effect value of −0.16, with a 95% BootCI of [−0.21, −0.12], which did not include 0. The indirect effect accounted for 61.54% of the total effect, indicating that rumination partially mediated the relationship between online upward social comparison and self-concept clarity.

Furthermore, self-compassion was introduced as a moderator to construct a moderated mediation model. Based on the hypothesized model, Model 14 was selected. The regression results showed that rumination significantly and negatively predicted self-concept clarity, and the interaction term between rumination and self-compassion significantly predicted self-concept clarity (*β* = 0.05, *t* = 2.41, *p* < 0.05) (see Table 3 for details). This suggests that the mediating effect of rumination in the relationship between online upward social comparison and self-concept clarity was moderated by self-compassion in the latter half of the pathway. To further understand the moderating role of self-compassion, a simple effect analysis was conducted by grouping participants into high and low self-compassion groups based on one standard deviation above and below the mean. The results indicated that compared to college freshmen with high self-compassion (*Slope* = −0.31, *t* = −9.79, *p* < 0.001), those with low self-compassion exhibited a steeper decline in self-concept clarity as rumination increased (*Slope* = −0.41, *t* = −11.16, *p* < 0.001). The simple slope analysis figure can be found in Supplementary Materials Figure S2.

The impact of online downward social comparison on self-concept clarity was finally tested. Self-concept clarity was designated as the dependent variable, online downward social comparison as the independent variable, and rumination as the mediator. A mediation analysis (Model 4) revealed that online downward social comparison had a significant negative effect on self-concept clarity (*β* = −0.24, *p* < 0.001). After accounting for rumination, this effect remained significant but was attenuated (*β* = −0.18, *p* < 0.001). Online downward social comparison positively predicted rumination (*β* = 0.13, *p* < 0.001), while rumination negatively predicted self-concept clarity (*β* = −0.47, *p* < 0.001). The indirect mediation effect was −0.06, with a 95% BootCI of [−0.10, −0.03], excluding 0. This indirect effect accounted for 25.00% of the total effect, confirming the partial mediating role of rumination in the relationship between online downward social comparison and self-concept clarity.

Next, self-compassion was incorporated as a moderator in a moderated mediation model (Model 14). The results indicated that rumination remained a significant negative predictor of self-concept clarity, and the interaction between rumination and self-compassion was also significant (*β* = 0.06, *t* = 2.72, *p* < 0.01; see Table 4). This suggests that self-compassion moderated the latter stage of the mediation pathway—specifically, the effect of rumination on self-concept clarity. To probe this moderation effect, self-compassion was divided into high and low groups (±1 SD from the mean). A simple slope analysis demonstrated that for students with low self-compassion, the negative association between rumination and self-concept clarity was stronger (*Slope* = −0.41, *t* = −11.50, *p* < 0.001) compared to those with high self-compassion (*Slope* = −0.30, *t* = −9.52, *p* < 0.001). The simple slope analysis figure can be found in Supplementary Materials Figure S3.

Furthermore, this study cross-validated the original SPSS 27.0 output against bootstrap estimates, confirming that the primary regression coefficients remained stable (with only minor variations in standard errors), thereby supporting the robustness of the proposed model.

## 4. Discussion

This study, based on social comparison theory (Festinger, 1954), employed a cross-sectional design to reveal the negative predictive effect of online social comparison (OSC) on self-concept clarity (SCC) among university freshmen and its underlying mechanisms. The results supported all three hypotheses: OSC significantly negatively predicted SCC (H1); rumination partially mediated the relationship between OSC and SCC (H2); and self-compassion buffered the negative impact of rumination on SCC (H3). Additionally, this study further distinguished the impact of the direction of online social comparison (upward vs. downward) on self-concept clarity. The findings indicated that both upward and downward social comparisons were significantly negatively correlated with SCC, but in the case of upward comparison, rumination fully mediated the relationship, while in downward comparison, rumination only partially mediated the effect. These findings enhanced the understanding of the relationship between online social comparison and self-concept in the digital age.

First, this study found a significant negative correlation between online social comparison and self-concept clarity among university freshmen, meaning that the more frequently students engage in online social comparison, the lower their self-concept clarity is. This finding aligns with previous empirical research (Butzer & Kuiper, 2006; Petre, 2021; Vartanian & Dey, 2013). It supports the “self-concept fragmentation hypothesis”—for individuals with unstable self-concepts, the use of social media may weaken their self-awareness, leading to lower self-concept clarity (Appel et al., 2018). Social media platforms, by presenting highly idealized and diverse self-representations (Vogel et al., 2014), unintentionally encourage individuals to engage in continuous social comparison (Fardouly et al., 2015). For university freshmen, who are in the identity exploration stage, exposure to vast and heterogeneous information online (such as academic achievements, appearance, social activity, etc.) makes their self-evaluation more susceptible to external influences, increasing the risk of difficulty in self-concept integration (Appel et al., 2018).

Second, our findings reveal the underlying mechanism through which online social comparison affects self-concept clarity, showing that online social comparison negatively influences self-concept clarity (SCC) through rumination. Research indicates that the pervasive and extreme nature of online social comparison easily triggers negative self-evaluations and negative emotions (Midgley et al., 2021; Vogel et al., 2014; Feinstein et al., 2013). Particularly for university freshmen, when they encounter information about others with a positive bias, they are more likely to perceive others as more successful and happier than themselves, which often distorts reality (Chou & Edge, 2011; Rosenberg & Egbert, 2011). This positively biased cognition threatens these students’ self-esteem, leading to increased rumination. Moreover, the unique nature of the university environment may amplify this process. University freshmen are in a new environment filled with diverse evaluative standards, where they compare not only academic achievements but also broader abilities, social relationships, and other personal traits that require time to develop. Therefore, when faced with a vast amount of multidimensional comparison information on social media, university freshmen may reflect more frequently on their own shortcomings and flaws. This self-centered negative thinking, namely rumination, ultimately undermines their self-acceptance and self-concept integration, leading to a decrease in self-concept clarity (Diehl & Hay, 2011). These findings are consistent with studies indicating the detrimental effects of online social comparisons on individuals (e.g., Yoon et al., 2019).

Another major finding of this study is that the mediating effect of rumination between online social comparisons and self-concept clarity (the second half path) is moderated by self-compassion. Specifically, compared to individuals with low levels of self-compassion, those with high levels of self-compassion experience a significant reduction in the negative impact of rumination on self-concept clarity.

This finding aligns with previous research demonstrating the protective role of self-compassion (Leary et al., 2007; Neff & Vonk, 2009). Neff (2003, 2023) highlighted that individuals high levels of self-compassion tend to approach themselves with greater openness and kindness. Thus, they not only focus on the positive aspects of their self but also embrace the negative aspects in a non-judgmental manner. This self-attentiveness improves self-awareness and self-acceptance, thereby enhancing self-concept clarity (Leary et al., 2007; Neff, 2003, 2023). Empirical studies have also shown that individuals with low self-compassion tend to magnify their thoughts and emotions of suffering, exhibiting more negative cognitive biases, negative emotions, and rumination. In contrast, individuals with higher levels of self-compassion exhibit the opposite tendency (Bian, 2019). Additionally, individuals with high self-compassion are more likely to employ positive coping strategies when confronted with negative events (Allen & Leary, 2010; Neff et al., 2005). Therefore, when engaging in rumination due to social comparisons, those with high self-compassion are less likely to remain trapped in passive rumination and worry. Instead, they actively reframe negative information and seek ways to regulate their emotions, thereby buffering the negative effects of rumination.

Finally, this study further investigated the effects of different directions of online social comparison (upward vs. downward) on self-concept clarity (SCC) among first-year university students and revealed the differentiated mediating role of rumination. The results showed that both online upward social comparison (OUSC) and online downward social comparison (ODSC) were significantly negatively associated with self-concept clarity. Regarding OUSC, this finding is consistent with a substantial body of previous research, which has demonstrated that upward social comparisons in online contexts often have detrimental effects on individuals’ self-perceptions (Midgley et al., 2021; Butzer & Kuiper, 2006).

However, the finding that ODSC also impairs SCC contradicts traditional social comparison theory, which posits that downward comparison functions as an adaptive strategy to maintain a positive self-evaluation and enhance self-esteem (Wills, 1981). Nevertheless, the negative effects of ODSC are not an isolated phenomenon and align with more recent empirical findings. For example, Laker and Waller (2022) found that for women, both upward and downward social comparisons conducted online were associated with poorer body image and self-perception. A recent meta-analysis by Bonfanti et al. (2025) also suggested that frequent social media use is associated with a stronger tendency to engage in social comparisons, which in turn may exacerbate body image concerns and problematic eating behaviors. These findings echo Collins’ (1996) earlier proposition that excessive engagement in social comparison may, in itself, lead to negative self-evaluations.

One possible explanation for this phenomenon is that frequent social comparisons may prompt individuals to rely excessively on external reference points to define and evaluate the self, rather than drawing upon an internal, stable self-concept. As Butzer and Kuiper (2006) noted, when individuals use information about others as the primary foundation for self-evaluation, their self-perceptions become highly susceptible to fluctuations in external input, thereby diminishing self-concept clarity.

Moreover, rumination emerged as a key mediating variable in the relationship between online social comparison and self-concept clarity. Specifically, rumination fully mediated the relationship between upward social comparison and SCC, whereas in the case of downward social comparison, it served only as a partial mediator. These findings suggest that the underlying mechanisms by which OUSC and ODSC influence self-concept clarity may differ.

### 4.1. Educational Implications

The findings of this study have important implications for the psychological health education practices of university freshmen. Given the spontaneous nature of online social comparison and its potential negative impact on self-concept clarity, merely advocating for a reduction in online social comparison behaviors may have limited effectiveness. Therefore, intervention strategies should shift their focus from external behavioral restrictions to the cultivation of internal psychological resources. This study identifies self-compassion as an important psychological protective factor that can effectively buffer the negative impact of rumination that is triggered by online social comparison on self-concept clarity.

In practical terms, universities could systematically integrate self-compassion training into existing psychological health education frameworks. Specific measures could include (1) adding a self-compassion module to general psychological health courses; (2) offering “Mindful Self-Compassion” (MSC) workshops for students in need; (3) promoting reflective writing exercises incorporating the principles of self-compassion. Empirical studies have shown that these interventions can effectively enhance individuals’ levels of self-compassion (Finlay-Jones et al., 2017; Galla, 2016; Toole & Craighead, 2016). By systematically implementing such training, universities can help students develop more adaptive emotional regulation strategies, thereby effectively mitigating the erosion of self-concept clarity that is caused by online social comparison and related rumination and promoting their long-term psychological health and personal development.

### 4.2. Limitations and Future Directions

Although this study provides valuable insights, several limitations warrant consideration. First, the cross-sectional design restricts the inference of causal relationships. Future research could adopt longitudinal designs or experimental manipulations to verify these relationships. Second, our sample comprised only Chinese college freshmen; however, the impact of cultural differences on the psychological effects of social comparison remains unclear. Consequently, future studies should investigate the moderating role of cultural background in more diverse, multicultural samples.

Third, this study did not collect data on participants’ place of residence. However, the participants’ place of residence may potentially influence the results of this study. Therefore, future research could include participants’ place of residence as a control variable for subsequent analysis.

Fourth, the exclusive reliance on self-reported questionnaires introduces potential social desirability and recall biases. To capture online social comparison experiences more accurately, future research could incorporate within-person, naturalistic assessment methods (e.g., ecological momentary assessment; Arigo et al., 2020). Fifth, our study focused exclusively on self-concept clarity, which only represents one aspect of self-concept. Previous research has indicated that online social comparisons can significantly impact other dimensions of self-concept, such as physical self-concept and appearance self-concept (Fardouly et al., 2015). Thus, future research could explore the mechanisms through which online social comparisons affect various dimensions and components of self-concepts. Finally, this study approached the topic from the perspective of online environments, and it remains unclear whether the same mechanisms apply to offline comparisons. Further research is needed to compare how online versus offline social comparison processes affect self-concept clarity.

## 5. Conclusions

This study revealed that online social comparison affected self-concept clarity among university freshmen through a moderated mediation model, specifically manifested as follows:

1. Partial Mediating Role of Rumination: Online social comparison not only directly impaired self-concept clarity but also indirectly reduced self-concept clarity by triggering individuals’ levels of rumination.
2. Protective Moderating Role of Self-Compassion: Self-compassion played a crucial buffering role in the latter part of the mediation pathway (i.e., the impact of rumination on self-concept clarity). For individuals with high levels of self-compassion, the negative effects of rumination were significantly diminished.

In summary, this study not only uncovered the psychological mechanisms through which online social comparison damaged self-concept clarity but also provided empirical support for universities to develop targeted psychological health intervention programs based on self-compassion.

## Figures and Tables

**Table 1:** Descriptive statistics and correlations for key variables.

Variable	*M*	*SD*	1	2	3	4	5	6	7	8
1. Gender	0.43	0.50	-							
2. Age	18.15	0.635	0.08 *	-						
3. OSC	2.68	0.73	−0.04	0.04	-					
4. OUSC	3.06	0.95	−0.08 *	0.04	0.86 ***	-				
5. ODSC	2.30	0.79	0.02	0.03	0.80 ***	0.39 ***	-			
6. SCC	3.01	0.73	0.03	−0.01	−0.31 ***	−0.26 ***	−0.24 ***	-		
7. Rumination	3.41	0.76	0.01	−0.02	0.31 ***	0.35 ***	0.13 ***	−0.50 ***	-	
8. Self-compassion	3.24	0.54	−0.01	−0.01	−0.31 ***	−0.32 ***	−0.19 ***	0.54 ***	−0.32 ***	-

Note: N = 975. OSC, online social comparison; OUSC, online upward social comparison; ODSC, online downward social comparison; SCC, self-concept clarity. * *p* < 0.05. *** *p* < 0.001.

**Table 2:** The relationship between online social comparisons and self-concept clarity: a moderated mediation model.

Independent Variable	Predictor Variable	*R* ^2^	*F*	*β*	*SE*	*t*	95% *CI*
Rumination	OSC	0.09	33.60 ***	0.31	0.03	10.02 ***	[0.25, 0.37]
	Gender			0.04	0.06	0.65	[−0.08, 0.16]
	Age			−0.05	0.05	−0.99	[−0.14, 0.05]
SCC	OSC	0.41	113.52 ***	−0.07	0.03	−2.72 **	[−0.13, −0.02]
	Rumination			−0.35	0.03	−12.79 ***	[−0.40, −0.29]
	Self-compassion			0.41	0.03	15.27 ***	[0.36, 0.46]
	Rumination × Self-compassion			0.05	0.02	2.40 *	[0.01, 0.09]
	Gender			0.06	0.05	1.17	[−0.04, 0.16]
	Age			−0.02	0.04	−0.43	[−0.09, 0.06]

Note: Standardized regression coefficients were reported. *N* = 975. OSC, online social comparison; SCC, self-concept clarity. * *p* < 0.05. ** *p* < 0.01. *** *p* < 0.001.

**Table 3:** The relationship between online upward social comparison and self-concept clarity: a moderated mediation model.

Independent Variable	Predictor Variable	*R* ^2^	*F*	*β*	*SE*	*t*	95% *CI*
Rumination	OUSC	0.13	47.46 ***	0.36	0.03	11.92 ***	[0.30, 0.42]
	Gender			0.08	0.06	1.23	[−0.05, 0.19]
	Age			−0.05	0.05	−1.12	[−0.15, 0.04]
SCC	OUSC	0.41	111.47 ***	0.01	0.03	0.28	[−0.05, 0.06]
	Rumination			−0.36	0.03	−13.21 ***	[−0.42, −0.31]
	Self-compassion			0.43	0.03	15.93 ***	[0.38, 0.48]
	Rumination × Self-compassion			0.05	0.02	2.41 *	[0.01, 0.09]
	Gender			0.07	0.05	1.32	[−0.03, 0.17]
	Age			−0.02	0.04	−0.57	[−0.10, 0.05]

Note: Standardized regression coefficients were reported. *N* = 975. OUSC, online upward social comparison; SCC, self-concept clarity. * *p* < 0.05. *** *p* < 0.001.

**Table 4:** The relationship between online downward social comparison and self-concept clarity: a moderated mediation model.

Independent Variable	Predictor Variable	*R* ^2^	*F*	*β*	*SE*	*t*	95% *CI*
Rumination	ODSC	0.02	6.06 ***	0.13	0.03	4.23 ***	[0.07, 0.20]
	Gender			0.01	0.06	0.12	[−0.12, 0.13]
	Age			−0.03	0.05	−0.65	[−0.13, 0.07]
SCC	ODSC	0.42	118.64 ***	−0.13	0.03	−5.05 ***	[−0.18, −0.08]
	Rumination			−0.35	0.03	−13.50 ***	[−0.40, −0.30]
	Self-compassion			0.41	0.03	15.57 ***	[0.36, 0.46]
	Rumination × Self-compassion			0.06	0.02	2.72 **	[0.02, 0.10]
	Gender			0.07	0.05	1.39	[−0.03, 0.17]
	Age			−0.02	0.04	−0.44	[−0.09, 0.06]

Note: Standardized regression coefficients were reported. *N* = 975. ODSC, online downward social comparison; SCC, self-concept clarity. ** *p* < 0.01. *** *p* < 0.001.

## Data Availability

The original contributions presented in this study are included in the article and Supplementary Material. Further inquiries can be directed to the corresponding authors.

## Supplementary Materials

The following supporting information can be downloaded at https://www.mdpi.com/article/10.3390/bs15070849/s1, Figure S1: The moderating effect of self-compassion in the Model 1 (The relationship between online social comparisons and self-concept clarity: a moderated mediation model); Figure S2: The moderating effect of self-compassion in the Model 2 (The relationship between online upward social comparison and self-concept clarity: a moderated mediation model); Figure S3: The moderating effect of self-compassion in the Model 3 (The relationship between online downward social comparison and self-concept clarity: a moderated mediation model).

## References

[B1-behavsci-15-00849] Aldao A., Nolen-Hoeksema S., Schweizer S. (2010). Emotion-regulation strategies across psychopathology: A meta-analytic review. Clinical Psychology Review.

[B2-behavsci-15-00849] Allen A. B., Leary M. R. (2010). Self-compassion, stress, and coping. Social and Personality Psychology Compass.

[B3-behavsci-15-00849] Appel M., Schreiner C., Weber S., Mara M., Gnambs T. (2018). Intensity of Facebook use is associated with lower self-concept clarity: Cross-sectional and longitudinal evidence. Journal of Media Psychology: Theories, Methods, and Applications.

[B4-behavsci-15-00849] Arigo D., Mogle J. A., Brown M. M., Pasko K., Travers L., Sweeder L., Smyth J. M. (2020). Methods to assess social comparison processes within persons in daily life: A scoping review. Frontiers in Psychology.

[B5-behavsci-15-00849] Arnett J. J. (2000). Emerging adulthood: A theory of development from the late teens through the twenties. American Psychologist.

[B6-behavsci-15-00849] Bai X. J., Liu X., Liu Z. J. (2013). The mediating effects of social comparison on the relations between achievement goal and academic self-effect: The evidence from the junior high school students. Journal of Psychological Science.

[B7-behavsci-15-00849] Bian X. H. (2019). The influence of self-compassion on negative bias and intervention. Doctoral dissertation.

[B8-behavsci-15-00849] Bigler M., Neimeyer G. J., Brown E. (2001). The divided self revisited: Effects of self-concept clarity and self-concept differentiation on psychological adjustment.

[B9-behavsci-15-00849] Bonfanti R. C., Melchiori F., Teti A., Albano G., Raffard S., Rodgers R., Lo Coco G. (2025). The association between social comparison in social media, body image concerns and eating disorder symptoms: A systematic review and meta-analysis. Body Image.

[B10-behavsci-15-00849] Brandenberg G., Ozimek P., Bierhoff H. W., Janker C. (2018). The relation between use intensity of private and professional SNS, social comparison, self-esteem, and depressive tendencies in the light of self-regulation. Behaviour & Information Technology.

[B11-behavsci-15-00849] Butzer B., Kuiper N. A. (2006). Relationships between the frequency of social comparisons and self-concept clarity, intolerance of uncertainty, anxiety, and depression. Personality and Individual Differences.

[B12-behavsci-15-00849] Campbell J. D. (1990). Self-esteem and clarity of the self-concept. Journal of Personality and Social Psychology.

[B13-behavsci-15-00849] Campbell J. D., Trapnell P. D., Heine S. J., Katz I. M., Lavallee L. F., Lehman D. R. (1996). Self-concept clarity: Measurement, personality correlates, and cultural boundaries. Journal of Personality and Social Psychology.

[B14-behavsci-15-00849] Chen J., Yan L. S., Zhou L. H. (2011). Reliability and validity of Chinese version of self-compassion scale. Chinese Journal of Clinical Psychology.

[B15-behavsci-15-00849] Chen S., Li X., Ye S. (2024). Self-concept clarity and meaning in life: A daily diary study in a collectivistic culture. Journal of Happiness Studies.

[B16-behavsci-15-00849] Chew L., Ang C. (2023). The relationship among quiet ego, authenticity, self-compassion and life satisfaction in adults. Current Psychology.

[B17-behavsci-15-00849] Chou H. G., Edge N. (2011). “They Are Happier and Having Better Lives than I Am”: The impact of using Facebook on perceptions of others’ lives. Cyberpsychology, Behavior, and Social Networking.

[B18-behavsci-15-00849] Collins R. L. (1996). For better or worse: The impact of upward social comparison on self-evaluations. Psychological Bulletin.

[B19-behavsci-15-00849] Diehl M., Hay E. L. (2011). Self-concept differentiation and self-concept clarity across adulthood: Associations with age and psychological well-being. The International Journal of Aging and Human Development.

[B20-behavsci-15-00849] Ding Q. (2017). The impact of social network sites use on adolescents’ self-evaluation: Base on social comparison theory. Doctoral dissertation.

[B21-behavsci-15-00849] Fardouly J., Diedrichs P. C., Vartanian L. R., Halliwell E. (2015). Social comparisons on social media: The impact of Facebook on young women’s body image concerns and mood. Body Image.

[B22-behavsci-15-00849] Fardouly J., Vartanian L. R. (2016). Social media and body image concerns: Current research and future directions. Current Opinion in Psychology.

[B23-behavsci-15-00849] Feinstein B. A., Hershenberg R., Bhatia V., Latack J. A., Meuwly N., Davila J. (2013). Negative social comparison on Facebook and depressive symptoms: Rumination as a mechanism. Psychology of Popular Media Culture.

[B24-behavsci-15-00849] Festinger L. (1954). A theory of social comparison processes. Human Relations.

[B25-behavsci-15-00849] Finlay-Jones A., Kane R., Rees C. (2017). Self-compassion online: A pilot study of an internet-based self-compassion cultivation program for psychology trainees. Journal of Clinical Psychology.

[B26-behavsci-15-00849] Galla B. M. (2016). Within-person changes in mindfulness and self-compassion predict enhanced emotional well-being in healthy, but stressed adolescents. Journal of Adolescence.

[B27-behavsci-15-00849] Gerson J., Plagnol A. C., Corr P. J. (2016). Subjective well-being and social media use: Do personality traits moderate the impact of social comparison on Facebook?. Computers in Human Behavior.

[B28-behavsci-15-00849] Gibbons F. X., Buunk B. P. (1999). Individual differences in social comparison: Development of a scale of social comparison orientation. Journal of Personality and Social Psychology.

[B29-behavsci-15-00849] Han X., Yang H. F. (2009). Chinese version of Nolen-Hoeksema ruminative responses scale (RRS) used in 912 college students: Reliability and validity. Chinese Journal of Clinical Psychology.

[B30-behavsci-15-00849] Harrington R., Loffredo D. A. (2010). Insight, rumination, and self-reflection as predictors of well-being. The Journal of Psychology.

[B31-behavsci-15-00849] Hayes A. F. (2013). Introduction to mediation, moderation, and conditional process analysis: A regression-based approach.

[B32-behavsci-15-00849] Higgins E. T. (1987). Self-discrepancy: A theory relating self and affect. Psychological Review.

[B33-behavsci-15-00849] Kim H., Callan M. J., Gheorghiu A. I., Skylark W. J. (2018). Social comparison processes in the experience of personal relative deprivation. Journal of Applied Social Psychology.

[B34-behavsci-15-00849] Laker V., Waller G. (2022). Does comparison of self with others influence body image among adult women? An experimental study in naturalistic settings. Eating and Weight Disorders-Studies On Anorexia Bulimia and Obesity.

[B35-behavsci-15-00849] Leary M. R., Tate E. B., Adams C. E., Batts Allen A., Hancock J. (2007). Self-compassion and reactions to unpleasant self-relevant events: The implications of treating oneself kindly. Journal of Personality and Social Psychology.

[B36-behavsci-15-00849] Lim M., Yang Y. (2015). Effects of users’ envy and shame on social comparison that occurs on social network services. Computers in Human Behavior.

[B37-behavsci-15-00849] Liu Q. Q., Niu G. F., Fan C. Y., Zhou Z. K. (2017). Passive use of social network site and its relationships with self-esteem and self-concept clarity: A moderated mediation analysis. Acta Psychologica Sinica.

[B38-behavsci-15-00849] Lockwood P., Shaughnessy S. C., Fortune J. L., Tong M. (2012). Social Comparisons in Novel Situations. Personality and Social Psychology Bulletin.

[B39-behavsci-15-00849] Lyubomirsky S., Tkach C. (2003). The consequences of dysphoric rumination. Depressive rumination.

[B40-behavsci-15-00849] MacBeth A., Gumley A. (2012). Exploring compassion: A meta-analysis of the association between self-compassion and psychopathology. Clinical Psychology Review.

[B41-behavsci-15-00849] Marciano L., Lin J., Sato T., Saboor S., Viswanath K. (2024). Does social media use make us happy? A meta-analysis on social media and positive well-being outcomes. SSM-Mental Health.

[B42-behavsci-15-00849] McComb C. A., Weidman A. C., Rogers M. L. (2023). A meta-analysis of the effects of upward social comparisons on social media on well-being. Journal of Applied Social Psychology.

[B43-behavsci-15-00849] Meier A., Gilbert A., Börner S., Possler D. (2020). Instagram inspiration: How upward comparison on social network sites can contribute to well-being. Journal of Communication.

[B44-behavsci-15-00849] Meier A., Schäfer S. (2018). The positive side of social comparison on social network sites: How envy can drive inspiration on Instagram. Cyberpsychology, Behavior, and Social Networking.

[B45-behavsci-15-00849] Midgley C., Thai S., Lockwood P., Kovacheff C., Page-Gould E. (2021). When every day is a high school reunion: Social media comparisons and self-esteem. Journal of Personality and Social Psychology.

[B46-behavsci-15-00849] Miyagawa Y. (2024). Self-compassion promotes self-concept clarity and self-change in response to negative events. Journal of Personality.

[B47-behavsci-15-00849] Neff K. D. (2003). The development and validation of a scale to measure self-compassion. Self and Identity.

[B48-behavsci-15-00849] Neff K. D. (2023). Self-compassion: Theory, method, research, and intervention. Annual Review of Psychology.

[B49-behavsci-15-00849] Neff K. D., Hsieh Y. P., Dejitterat K. (2005). Self-compassion, achievement goals, and coping with academic failure. Self and Identity.

[B50-behavsci-15-00849] Neff K. D., Vonk R. (2009). Self-compassion versus global self-esteem: Two different ways of relating to oneself. Journal of Personality.

[B51-behavsci-15-00849] Nesi J., Prinstein M. J. (2015). Using social media for social comparison and feedback-seeking: Gender and popularity moderate associations with depressive symptoms. Journal of Abnormal Child Psychology.

[B52-behavsci-15-00849] Niu X., Gou L., Han Y., Zhou X., Wang J. (2024). Self-concept clarity and envy as mediators between upward social comparison on social networking sites and subjective well-being. British Journal of Developmental Psychology.

[B53-behavsci-15-00849] Nolen-Hoeksema S., Morrow J. (1991). A prospective study of depression and posttraumatic stress symptoms after a natural disaster: The 1989 Loma Prieta earthquake. Journal of Personality and Social Psychology.

[B54-behavsci-15-00849] Nolen-Hoeksema S., Parker L. E., Larson J. (1994). Ruminative coping with depressed mood following loss. Journal of Personality and Social Psychology.

[B55-behavsci-15-00849] Nolen-Hoeksema S., Wisco B. E., Lyubomirsky S. (2008). Rethinking rumination. Perspectives on Psychological Science.

[B56-behavsci-15-00849] Orben A., Meier A., Dalgleish T., Blakemore S. (2024). Mechanisms linking social media use to adolescent mental health vulnerability. Nature Reviews Psychology.

[B57-behavsci-15-00849] Ouwerkerk J. W., Johnson B. K. (2016). Motives for online friending and following: The dark side of social network site connections. Social Media + Society.

[B58-behavsci-15-00849] Petre C. E. (2021). The relationship between Internet use and self-concept clarity: A systematic review and meta-analysis. Cyberpsychology: Journal of Psychosocial Research on Cyberspace.

[B59-behavsci-15-00849] Podsakoff P. M., MacKenzie S. B., Lee J.-Y., Podsakoff N. P. (2003). Common method biases in behavioral research: A critical review of the literature and recommended remedies. Journal of Applied Psychology.

[B60-behavsci-15-00849] Robinson M. S., Alloy L. B. (2003). Negative cognitive styles and stress-reactive rumination interact to predict depression: A prospective study. Cognitive Therapy and Research.

[B61-behavsci-15-00849] Rosenberg J., Egbert N. (2011). Online impression management: Personality traits and concerns for secondary goals as predictors of self-presentation tactics on Facebook. Journal of Computer-Mediated Communication.

[B62-behavsci-15-00849] Stapleton P., Luiz G., Chatwin H. (2017). Generation validation: The role of social comparison in use of Instagram among emerging adults. Cyberpsychology, Behavior and Social Networking.

[B63-behavsci-15-00849] Şimşek Ö. F. (2013). The relationship between language use and depression: Illuminating the importance of self-reflection, self-rumination, and the need for absolute truth. The Journal of General Psychology.

[B64-behavsci-15-00849] Toole A. M., Craighead L. W. (2016). Brief self-compassion meditation training for body image distress in young adult women. Body Image.

[B65-behavsci-15-00849] Trapnell P. D., Campbell J. D. (1999). Private self-consciousness and the five-factor model of personality: Distinguishing rumination from reflection. Journal of Personality and Social Psychology.

[B66-behavsci-15-00849] Treynor W., Gonzalez R., Nolen-Hoeksema S. (2003). Rumination reconsidered: A psychometric analysis. Cognitive Therapy and Research.

[B67-behavsci-15-00849] Van Dijk M. P. A., Branje S., Keijsers L., Hawk S. T., Hale W. W. R., Meeus W. (2014). Self-concept clarity across adolescence: Longitudinal associations with open communication with parents and internalizing symptoms. Journal of Youth and Adolescence.

[B68-behavsci-15-00849] Vartanian L. R., Dey S. (2013). Self-concept clarity, thin-ideal internalization, and appearance-related social comparison as predictors of body dissatisfaction. Body image.

[B69-behavsci-15-00849] Verduyn P., Gugushvili N., Massar K., Täht K., Kross E. (2020). Social comparison on social networking sites. Current Opinion in Psychology.

[B70-behavsci-15-00849] Vogel E. A., Rose J. P., Okdie B. M., Eckles K., Franz B. (2015). Who compares and despairs? The effect of social comparison orientation on social media use and its outcomes. Personality and Individual Differences.

[B71-behavsci-15-00849] Vogel E. A., Rose J. P., Roberts L. R., Eckles K. (2014). Social comparison, social media, and self-esteem. Psychology of Popular Media Culture.

[B72-behavsci-15-00849] Wang J. L., Wang H. Z., Gaskin J., Hawk S. (2017). The mediating roles of upward social comparison and self-esteem and the moderating role of social comparison orientation in the association between social networking site usage and subjective Well-being. Frontiers in Psychology.

[B73-behavsci-15-00849] Watkins E. R. (2008). Constructive and unconstructive repetitive thought. Psychological Bulletin.

[B74-behavsci-15-00849] Weinstein E. (2017). Adolescents’ differential responses to social media browsing: Exploring causes and consequences for intervention. Computers in Human Behavior.

[B75-behavsci-15-00849] Wen Z. L., Ye B. J. (2014). Different methods for testing moderated mediation models: Competitors or backups?. Acta Psychologica Sinica.

[B76-behavsci-15-00849] Willis K. D., Burnett H. J. (2016). The power of stress: Perceived stress and its relationship with rumination, self-concept clarity, and resilience. North American Journal of Psychology.

[B77-behavsci-15-00849] Wills T. A. (1981). Downward comparison principles in social psychology. Psychological Bulletin.

[B78-behavsci-15-00849] Wood J. V. (1996). What is social comparison and how should we study it?. Personality and Social Psychology Bulletin.

[B79-behavsci-15-00849] Xiang G., Teng Z., Li Q., Chen H. (2023). Self-concept clarity and subjective well-being: Disentangling within- and between-person associations. Journal of Happiness Studies.

[B80-behavsci-15-00849] Yang C. C., Holden S. M., Carter M. D. K. (2018). Social media social comparison of ability (but not opinion) predicts lower identity clarity: Identity processing style as a mediator. Journal of Youth and Adolescence.

[B81-behavsci-15-00849] Yoon S., Kleinman M., Mertz J., Brannick M. (2019). Is social network site usage related to depression? A meta-analysis of Facebook-depression relations. Journal of Affective Disorders.

[B82-behavsci-15-00849] Zhang Z., Yuan K. H. (2018). Practical statistical power analysis using Webpower and R.

