# Supplementary figures and images for: Do College Freshmen Who Engage More in Online Social Comparison Tend to Be More Confused About Themselves? The Roles of Rumination and Self-Compassion
Source: Behav Sci (Basel). 2025 Jun 24;15(7):849. doi: 10.3390/bs15070849 (PMC12292234; doi:10.3390/bs15070849)

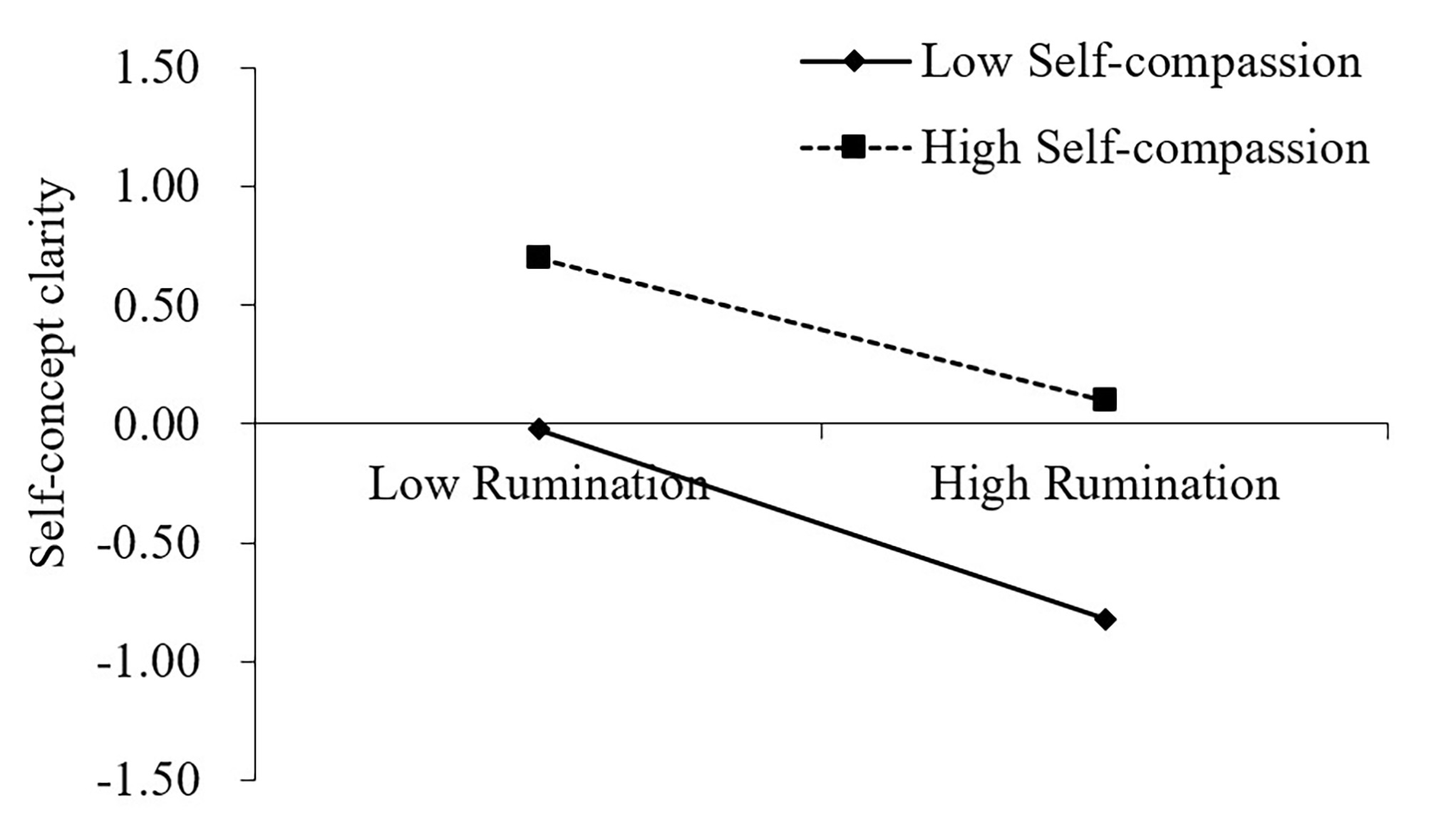

Supplement: Supplementary file 1 [file behavsci-15-00849-s001.zip › Figure S1.jpg]

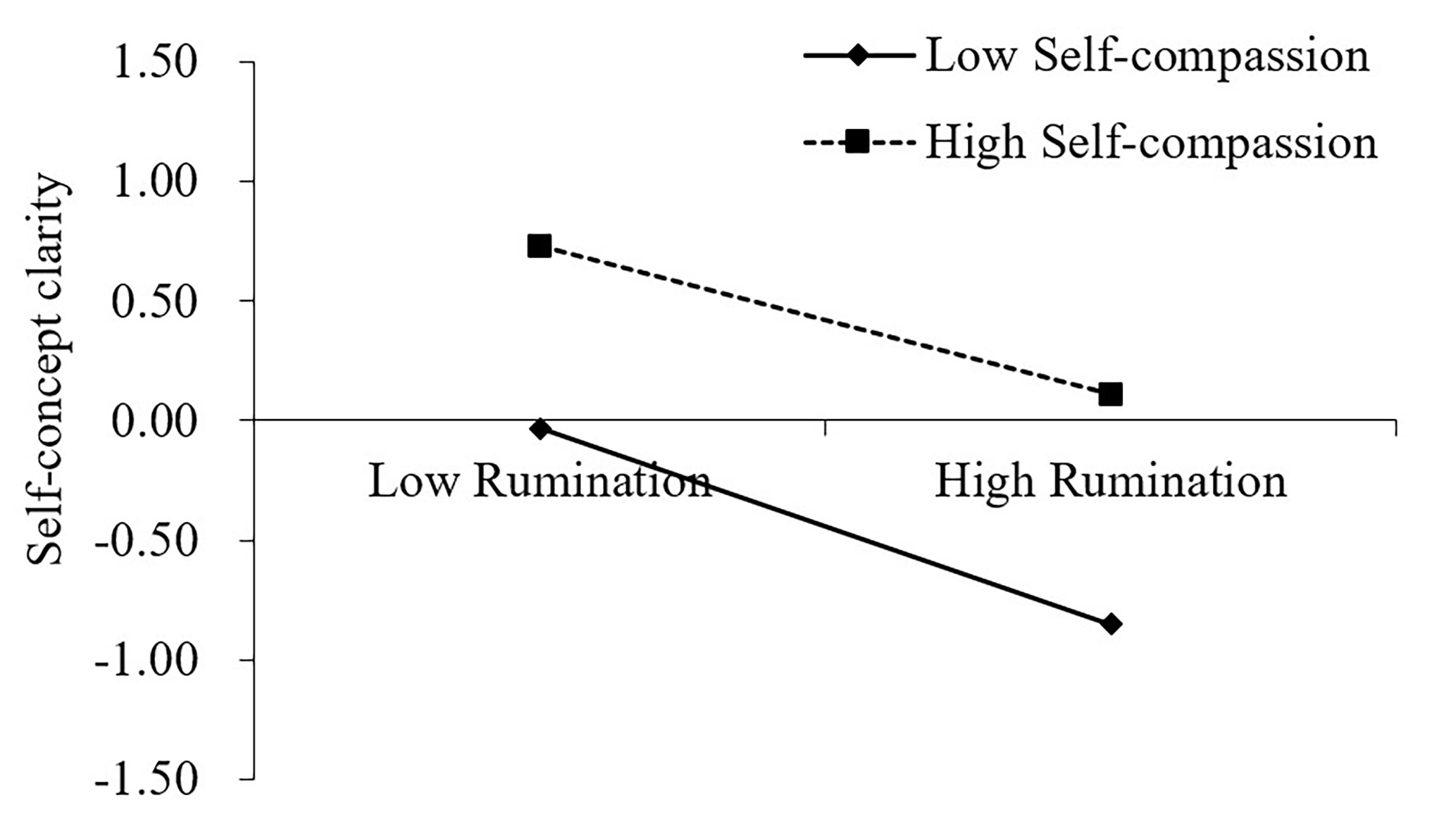

Supplement: Supplementary file 1 [file behavsci-15-00849-s001.zip › Figure S2.jpg]

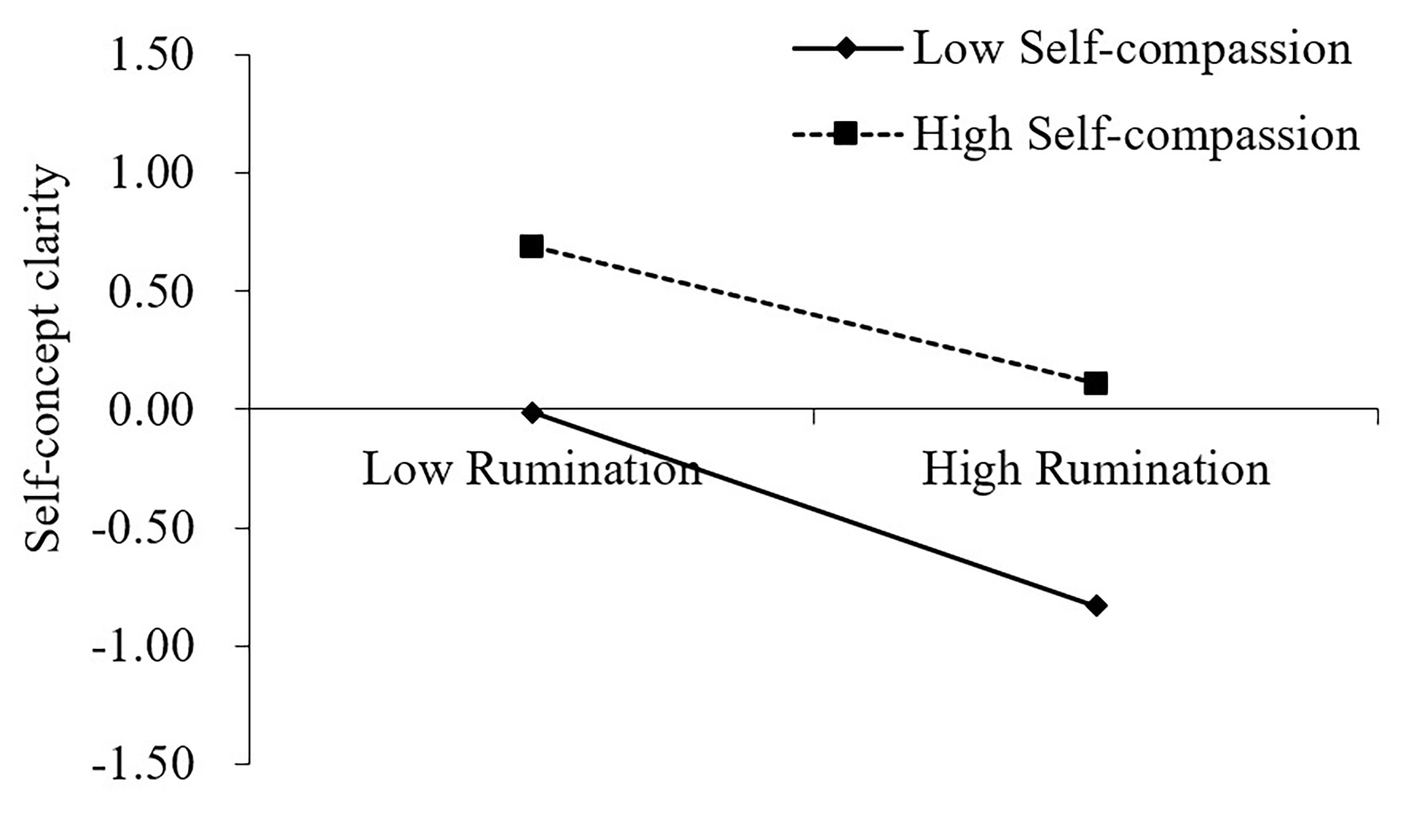

Supplement: Supplementary file 1 [file behavsci-15-00849-s001.zip › Figure S3.jpg]
